# An Ultrasensitive Biomimetic Optic Afferent Nervous System with Circadian Learnability

**DOI:** 10.1002/advs.202309489

**Published:** 2024-03-11

**Authors:** Kaiyang Wang, Shuhui Ren, Yunfang Jia, Xiaobing Yan

**Affiliations:** ^1^ College of Electronic Information and Optical Engineering Nankai University Tianjin 300071 P. R. China; ^2^ Key Laboratory of Brain‐Like Neuromorphic Devices and Systems of Hebei Province College of Electron and Information Engineering Hebei University Baoding 071002 P. R. China

**Keywords:** circadian rhythm, heterojunction, neurotransmitter, optic afferent nervous system, synapse

## Abstract

The optic afferent nervous system (OANS) plays a significant role in generating vision and circadian behaviors based on light detection and signals from the endocrine system. However, the bionic simulation of this photochemically mediated behavior is still a challenge for neuromorphic devices. Herein, stimuli of neurotransmitters at ultralow concentrations and illumination are coupled to artificial synapses with the aid of biofunctionalized heterojunction and tunneling to successfully simulate a circadian neural response. Furthermore, the mechanisms underlying the photosensitive synaptic current in response to stimuli are described. Interestingly, this OANS is demonstrated to be capable of mimicking normal and abnormal circadian learnability by combining the measured synaptic current with a three‐layer spike neural network. Strong theoretical and experimental evidence, as well as applications, are provided for the proposed biomimetic OANS to demonstrate that it can reproduce biological circadian behavior, thus establishing it as a promising candidate for future neuromorphic intelligent robots.

## Graphical abstract

A biomimetic optic afferent nervous system is proposed to mimic circadian rhythm by constructing biochemical and photosensitive interfaces on the traditional MXene synapse. The experimental and theoretical studies are elaborately conducted to demonstrate its sensitivities for light and serotonin. It can not only simulate the synaptic plasticity modulated by serotonin and light but also the circadian learning behaviors.

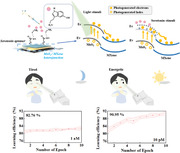

## 1 Introduction

The optic nerve system is a unique part of the afferent nervous system (ANS) that can not only transmit external images to the brain but also participate in the neural activity related to the circadian rhythm, a biological clock that alters with sunrise and sunset.^[^ ^1^, ^2^, ^3^, ^4^, ^5^, ^6^ ^]^ Optic neurological studies have demonstrated that the response of the optic afferent nervous system (OANS) to light is an indispensable factor in maintaining circadian rhythm, and abnormal rhythm may lead to fatigue, depression, and even loss of learnability and memory.^[^ ^7^, ^8^, ^9^, ^10^ ^]^ From this perspective, as the main carrier of neural signal transmission in OANS, the synapse not only generates vision but also plays an important role in regulating the physiological behavior of circadian rhythm.^[^ ^11^, ^12^, ^13^ ^]^ To date, artificial photosensitive synapses have caused a wide upsurge in research, and the development of low‐power and high‐speed photosensitive synaptic devices lays the foundation for researches on biomimetic vision in light perception.^[^ ^14^, ^15^ ^]^ Moreover, by using artificial optic synapses, light‐driven neural responses, visual senses, and reflex behaviors have been successfully simulated.^[^ ^16^, ^17^, ^18^ ^]^ However, it still remains a challenge for current photosensitive artificial synapses to simultaneously accomplish visual perception and circadian functions, which are believed to be regulated by both external light and internal neurotransmitters.^[^ ^10^ ^]^ If a photochemistry dual‐functional artificial OANS can be constructed at the level of an integrated and minimized neuromorphic chip, it will help to create a visual system closer to the biological optic neuropathy and promote new developments in the field of bionic perception.

**Figure 1:**
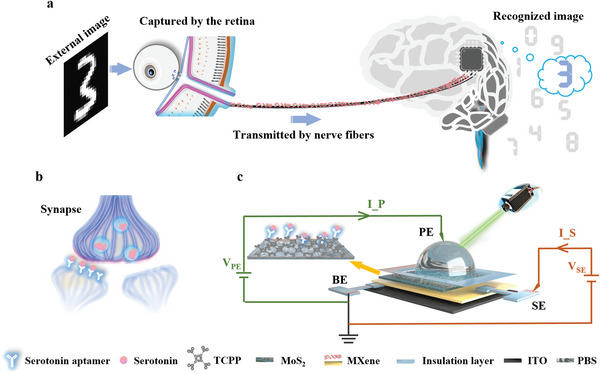
Schematic diagram of the biological and artificial optic afferent nervous system (OANS). a) In biological OANS, the external image is captured by the retina and then transformed by optical synapse from the optical form to the neurological electric form which is transmitted by nerve fibers to the brain and recognized as visual images. b) In OANS, the biological synapse plays an important role in regulating the circadian neural response because its synaptic plasticity can be modulated by both ambient light and endocrine neurotransmitters (serotonin as a proof‐of‐concept). c) The schematic of the current measured path of the proposed OANS device. The perception electrode (PE) is the suspended Pt electrode in PBS solution, synapse electrode (SE) is the Ag electrode on the MXene layer. The bottom electrode (BE) is the ITO electrode.

Optic synapses are the connections between the retina and brain, as depicted in **Figure** **1**a. According to the conventional theory, they are only responsible for transforming external image signals from the optical form to the neurological electric form, which are reconstructed as visual images in the brain. However, modern optic neurology and neurochemistry indicate that visual perception is only a part of the OANS, as optic synapses can cooperate with neurotransmitters to regulate the behaviors with biological rhythms.^[^ ^19^, ^20^, ^21^ ^]^ For example, the learnability and memory of visual stimuli (such as the image in Figure 1a) are dually controlled by environmental light and diffused neurotransmitters (such as serotonin) from the suprachiasmatic nucleus of the hypothalamus (Figure 1b). For the same image, the intensity of the neural response produced by OANS to the brain is determined not only by light but also by neurotransmitters. Therefore, equal attention should be paid to the effect of neurotransmitters on synaptic plasticity in studies of artificial OANS. In our previous work, attempts to simulate the neurotransmitter (ACh) modulated neuronal response, as well as to detect and record injured responses, were accomplished by introducing an ACh receptor (AChR) cross‐linked third terminal on the traditional two‐terminal artificial synapse.^[^ ^22^ ^]^ Moreover, a biologically hybrid synapse with the neurotransmitter plasticity was reported, in which PEDOT: PSS was used as the functional layer, and the dynamic responses of nerve cells to the neurotransmitter were simulated through the redox characteristics of dopamine.^[^ ^23^ ^]^ The electrical, chemical, and optical properties of organic‐based neuromorphic devices can be easily controlled through molecular design or surface functionalization, enabling the multifunctionality of electronic/ion conduction‐based neuromorphic devices to be realized.^[^ ^24^ ^]^ Recently, a synaptic transistor was developed to simultaneously imitate the optic and chemical double‐controlled neural response. In this case, the reproduction of the dopaminergic signaling was affected by rhythmic illumination, which contributed to the emerging bionic neuromorphic system.^[^ ^10^ ^]^ Moreover, the effort to mimic visual memory with the use of the synaptic field‐effect transistors demonstrated that the memory retention levels to optical inputs could be modulated by gate voltages, which introduced a new paradigm to realize bionic optic nerve systems.^[^ ^25^ ^]^ Overall, artificial optic synapses are considered the key components in OANS, and recent contributions can fulfill the tasks of visual perception, visual memory, or light‐rhythmic plasticity. However, advanced neurology works highlight that daily rhythmically fluctuating memory and learnability are closely related to circadian rhythm‐related neurotransmitters, artificial optic synapses that mimic neurotransmitter‐triggered circadian rhythmic visual memory or learnability are seldom found. If the circadian behavior modulated by neurotransmitters can be imitated, it will broaden the functionality of biomimetic OANSs and make a significant impact on the boom in bionic robots.

In this work, a biomimetic OANS device based on the MXene/MoS_2_ heterojunction and neurotransmitter‐mediated MXene synapse for imitating circadian behavior is proposed. The main operating principle of the system is graphically depicted in Figure 1c and briefly described here. First, the synaptic plasticity can be simulated using the MXene artificial synapse, which consists of an MXene layer sandwiched between the bottom electrode (ITO, named BE) and the synapse electrode (Ag, named SE). Second, the MXene/MoS_2_ heterojunction is constructed as the perception electrode (Pt, named PE), which is used to separate the photogenerated carriers and transform them into the synaptic current (I_S). Third, the surface of the MoS_2_ film is modified by tris (chloropropyl) phosphate (TCPP) and the aptamer of a neurotransmitter (serotonin is used as a proof‐of‐concept) anchored on TCPP to mimic neurochemical modulation. Therefore, synaptic plasticity can be modulated by controlled illumination and serotonin concentration, which are similar to ambient light and internal secretion. To demonstrate these dual modulations, the plasticity of the proposed biomimetic photochemistry‐sensitive synapses is thoroughly examined by measuring I_S and the resistive switching processes. Finally, a novel simulation of circadian learnability is conducted for the first time by applying the proposed devices in a three‐layer spiking neural network (SNN), which represents a bionic calculation that mimics the brain‐like reasoning process. The simulation of learning efficiency in normal and abnormal circadian rhythms under different optical and chemical conditions is conducted, which yields results similar to real circadian and endocrine changes. We believe that this work opens up a new pathway for developing artificial OANSs and realizing neuromorphic intelligent robots.

## 2 Results and Discussion

### 2.1 Impact of the Heterojunction on the MXene Synapse

**Figure 2:**
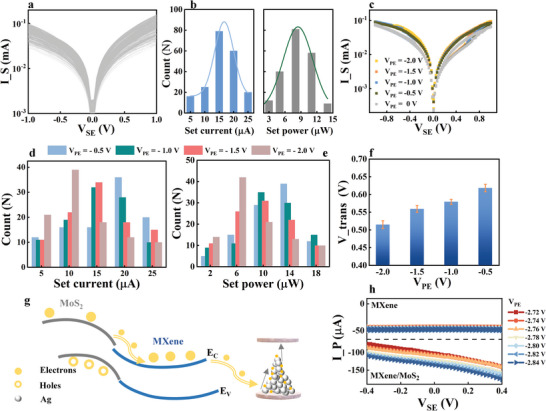
The basic electronic performance of as‐prepared MXene and MXene/MoS_2_ heterojunction integrated Ti_3_C_2_T_x_ artificial synapses. a) The 1000 times CV curves. b) The statistical analyses of Set current and Set power. c) The influences of V_PE_ on CV curves. d) Set current, and e) Set power under different V_PE_. f) The statistical analyses of the turning point voltages between Fowler‐Northeim tunneling and direct tunneling conduction modes under different V_PE_. g) The diagram of the conductive filament‐based working principle. h) The transfer characteristic curves of the devices without and with heterojunction, when V_PE_ is controlled from −2.72 to −2.84 V.

Few‐layer MXene, as a promising 2D electronic material, has been widely exploited in the fields of artificial synapses and sensors.^[^ ^26^, ^27^, ^28^, ^29^, ^30^, ^31^ ^]^ Here, to investigate its possibilities for fabricating the artificial optic synapse in OANS, we tested the electronic performances of the prepared MXene synapses under varied illumination and chemical environmental conditions. Schematic illustrations of the preparation processes and electrical circuit connection mode are provided in Figure S1 (Supporting Information) and Figure 1c. **Figure** **2**a shows the 1000 cyclic voltammetry (CV) curves of the prepared MXene synapse, and the confirmed excellent repeatability guarantees the reliability of subsequent tests. According to the CV curves in Figure 2a, statistical analyses of the Set current and Set power are given in Figure 2b. The Set current and Set power of the proposed MXene artificial synapse are concentrated ≈15 µA and 7 µW, respectively, and Gaussian fitting indicates that the distributions of the Set current and Set power are relatively centralized.^[^ ^32^ ^]^ The statistics for the high resistance state (HRS) and low resistance state (LRS) are shown in Figure S2a (Supporting Information), and the HRS/LRS ratio is ≈3.5. Further statistical analysis of the synaptic current ratio (I_LRS_/I_HRS_) was conducted, and the values exhibited a uniform Gaussian distribution, as shown in Figure S2b (Supporting Information). The asymptotic nonlinear CV curves (Figure S3, Supporting Information) indicate that with increased sweeping times, I_S in the positive and negative ranges increase and decrease, respectively, which indicates that the as‐prepared device shows a reliable “analog” switching behavior and can achieve synaptic plasticity and learning behavior as a memristor. Subsequently, to explore the influence of the MXene/MoS_2_ heterojunction on the synapse, different bias voltages were applied to the PE (V_PE_). As shown in Figure 2c, the changes of I_S can be found when different V_PE_s are applied, and the changes on the positive side are clearer than those on the negative side. Meanwhile, the enlarged CV curves (Figure S4, Supporting Information) indicate that the Set voltage decreases with negatively increased V_PE_. Additionally, the potential for low‐power application can be proven by statistical analyses of the Set current and Set power under different V_PE_s, as shown in Figure 2d,e. With the negatively increased V_PE_, the median values of the Set current are reduced from 20 to 10 µA, and those of the Set power are reduced from 14 to 7 µW. The electronic performances of similar devices are compared in Table S1 (Supporting Information). Refitted CV curves (under different V_PE_s) by using Fowler‐Northeim tunneling and direct tunneling conduction models are given in Figure S5 (Supporting Information). The transmission modes are changed with the swept voltages on the SE (named V_SE_), and the turning point is named V_trans_. When V_SE_ is larger than V_trans_, the Fowler‐Northeim tunneling mode carrier transmission overcomes the direct tunneling mode transmission; hence, I_S increased. The summarized V_trans_ values presented in Figure 2f are lowered with the increased negative V_PE_. Further explanation for this phenomenon is provided below. To understand the physical mechanisms of the V_PE_‐modulated synaptic features, additional theories are presented. First, the working mechanism of the MXene/MoS_2_ heterojunction originates from the energy band bending at the interface between MXene and MoS_2_, as shown in Figure 2g. The higher work function (5.15 eV) of MoS_2_ compared with the 2D MXene nanosheets (4.3 eV) allows electrons in the conduction band of MXene to be naturally injected into the MoS_2_, leaving positively charged ions. Conversely, holes on the MoS_2_ side will be injected into MXene and negative charges will be left. This spontaneous carrier migration caused by differences in work functions results in a downward bending of the energy band structure and the formation of a space charge region at the MXene/MoS_2_ heterojunction. In this region, the built‐in electric field can drive electrons to MXene and holes to MoS_2_. If there are no external stimuli, the two types of carrier movement will be in a dynamic equilibrium state. Then, according to the theory of conductive filament,^[^ ^33^, ^34^, ^35^ ^]^ when the SE is biased by a controlled V_SE_, the metal ions (Ag^+^) move toward the BE under the action of electric field force. During transport, silver ions react with electrons and are reduced to silver atoms; therefore, a conductive filament region is formed by the accumulated Ag atoms (as illustrated in Figure 2g). To confirm the production of Ag conductive filament in the resistance switching process, the results of electrode cutting and temperature dependence testing are provided in Figure S6 (Supporting Information). Unlike other regions of the MXene layer, there is a strong local electric field that has an attraction effect on the electrons adjacent to the conductive filament. When the electrons in the conduction band of MoS_2_ are injected into the MXene aided by the electric field, they are easily transported to the conductive filament region due to the Joule heat generated by the V_PE_ and the attraction of the Ag conductive filament. They can then participate in the reaction Ag^+^ + e ⇆ Ag and help enhance the synaptic current. Furthermore, the carrier transmission modes through the heterojunction, i.e., Fowler‐Northeim tunneling and direct tunneling are accounted to explain the effects of V_PE_, according to the literature.^[^ ^36^, ^37^, ^38^ ^]^ The gradually decreasing V_trans_ (0.63 V → 0.56 V → 0.54 V → 0.52 V) in Figure 2f means that the electrons in the MoS_2_ layer may pass through the heterojunction more easily with a negatively increased V_PE_, and then, more electrons may be transported to the conductive filament region and foster the reduction of Ag^+^. Therefore, it is reasonable to believe that the electrons injected from the MXene/MoS_2_ heterojunction are beneficial for the formation of conductive filaments. Additionally, our speculation about heterojunction‐enhanced I_S can also be confirmed from another point‐of‐view, by comparing the transfer characteristic curves of the proposed device without and with the MoS_2_ structure as shown in Figure 2h. The current through the PE (named I_P) indicates that there is a current flow across the interface of the device surface and phosphate‐buffered saline (PBS), and it may be enhanced by the MXene/MoS_2_ heterojunction, as illustrated by the values without (≈−48∼−44 µA) and with (≈−160∼−80 µA) the MXene/MoS_2_ heterojunction. This means that the existence of the MXene/MoS_2_ heterojunction contributes to the separation of carriers and allows more of them to enter the conductive filament region. In particular, the ability to separate carriers can be controlled by the bias voltage, as shown by the comparison of results measured by the OANS devices with and without the MXene/MoS_2_ heterojunction (Figure 2h).

### 2.2 Light‐Mediated Synaptic Plasticity

**Figure 3:**
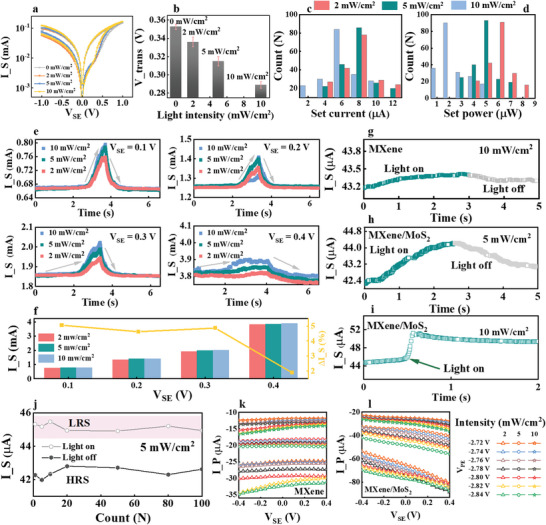
The photoelectric sensitive performance of the OANS device. a) The logarithmic *I*–*V* curves of the MXene Ti_3_C_2_T_x_ artificial synapse under different light intensities. b) The statistical analyses of the turning point of MXene Ti_3_C_2_T_x_ artificial synapse under different light intensities. The error bar is the standard deviation of test results measured by three individual devices. The statistical analyses of the Set current (c) and Set power (d) under different light intensities. e) I_S change curves accompanying the swept V_PE_ (0 V – −2V − 0 V, scanning with the arrow direction) under different V_SE_ (0.1, 0.2, 0.3, and 0.4 V) and light intensities (2, 5, and 10 mW cm^−2^). The I_S and synaptic current change rates (∆I_S) at each of the peak positions are summarized in (f). g) Dynamic I_S transient curve of the device without MXene/MoS_2_ heterojunction in response to 10 mW cm^−2^ light, V_SE_ = 0.1 V, V_PE_ = −2 V. h) Dynamic I_S transient curve of the device with MXene/MoS_2_ heterojunction in response to 5 mW cm^−2^ light, V_SE_ = 0.1 V, V_PE_ = −2 V. i) I_S dynamic transient curve, under the continuously controlled illumination (10 mW cm^−2^), V_SE_ = 0.1 V, V_PE_ = −2 V. j) The Statistical distributions of I_S under light on and off, respectively. (k) and (l) are the transfer characteristic curves of the devices without and with the MXene/MoS_2_ heterojunction, under different V_PE_ and light intensities.

The optic synapse is the conveyor in the OANS,^[^ ^39^, ^40^, ^41^ ^]^ and it is responsible for sensing the external light and semiquantitatively transforming it into an electronic neural signal. Therefore, to test the possibility of the proposed device as a biomimetic optic synapse in the OANS, its photo‐sensitivities were measured in this section. According to the optical absorption characteristics of the synthesized MXene and optical features of MoS_2_ (Figures S7–S11, Supporting Information), a laser with a wavelength of 550 nm was chosen and irradiated on the area of PBS buffer‐coated surface as diagramed in Figure 1c. When MoS_2_ absorbs enough energy, extra electron–hole pairs can be generated. Although they are unbalanced and easily recombined, in the proposed OANS device, they can be separated at the junction of MXene and MoS_2_ with the aid of the space charge region. As a result, more photogenerated electrons may be transported to the conductive filament region and enhance I_S. To examine the influence of light, its intensities were controlled by adjusting the laser power from 0 to 10 mW cm^−2^. When the powers were controlled at 0, 2, 5, and 10 mW cm^−2^ and the V_PE_ was −2 V, CV curves were recorded (as shown in **Figure** **3**a). The contours of the curves changed slightly. To reveal the potential effects of light on the synapse, CV curves were refitted by using Fowler‐Northeim tunneling and direct tunneling conduction models (Figure S12, Supporting Information), and the summarized V_trans_ values are presented in Figure 3b. When the light intensity increases from 0 to 10 mW cm^−2^, V_trans_ changes from 0.35 to 0.29 V, which means that the electron current in the Fowler‐Northeim tunneling mode is gradually increased by enhancing the illumination. Since all the other working conditions are unchanged, such an increase should be attributed to the photogenerated carriers. The total effect of light on the synapse can be explained as follows: the excited electron–hole pairs are separated by the MXene/MoS_2_ heterojunction; then, the separated photogenerated electrons are injected into the MXene and participate in the conductive filament formation, thus I_S is formed. The statistical analyses of Set current and Set power under different light intensities are shown in Figure 3c,d. With increasing light intensity, the mode value of the Set current is reduced from 8 to 6 µA, and the Set power is reduced from 6 to 2 µW. These values demonstrate that increased light intensity can further reduce the power consumption of the MXene artificial synapse in SET processes. The results of the uniform Gaussian fitting are shown in Figures S13 and S14 (Supporting Information). The variations in I_S at each light intensity and each V_SE_ demonstrate that the ability of the heterojunction to separate photogenerated carriers is adjusted by the negative bias voltage, and the maximum value is shown by the peaks in Figure 3e. Summaries of these peaks and the synaptic current change rate (∆I_S) with V_PE_ at these points are presented in Figure 3f. ∆I_S is defined as the change rate of the peak value relative to the initial value after the tactile experiment ((I_S max – I_S min) /I_S min) × 100%, in which I_S max and I_S min are the maximum and minimum synaptic currents, respectively. Higher I_S values are found in the group of V_SE_ = 0.4 V; however, the larger ∆I_S belongs to V_SE_ = 0.1 V. Therefore, V_SE_ = 0.1 V is used for subsequent tests. More evidences are presented in Figure 3g,h to clarify the active role of the MXene/MoS_2_ heterojunction in modulating I_S. These results show the light responses of similar devices without and with the MXene/MoS_2_ heterojunction, respectively. For the device without the MXene/MoS_2_ heterojunction (Figure 3g), the I_S curve is almost flat, and no fluctuations are observed at the conjunction of the black (light on) and gray (light off) data points. In contrast, only under the 5 mW cm^−2^, the photosensitivity of the device with MXene/MoS_2_ is clearly shown by the I_S curve in Figure 3h. Other I_S curves without and with the MXene/MoS_2_ heterojunction under different light intensities are presented in Figures S15 and S16 (Supporting Information). And the cyclic response results to repeated light stimuli (5 mW cm^−2^) of the OANS device can be found in Figure S17 (Supporting Information). In addition, a dynamic transient I_S test was executed (1) when light is suddenly turned on and off, I_S jumps up and down, as shown in Figure 3i; (2) when light is repetitively turned on and off, I_S continuously changes (Figure S18, Supporting Information). These phenomena correspond to the electroforming model.^[^ ^42^ ^]^ Subsequently, the 100‐cycle endurance tests of I_S were performed under 5 mW cm^−2^ light intensity. As presented in Figure 3j, I_S is stable in two states, which is believed to be conducive to memory simulation. Additional evidence supporting the function of the heterojunction is provided by the transfer characteristic curves in Figure 3k,l, and Figure S19 (Supporting Information), which were measured by the devices without and with the MXene/MoS_2_ heterojunction, respectively, when V_PE_ was controlled at −2.48 to −2.72 V and the light intensities were 2, 5 and 10 mW cm^−2^. The enhanced I_S can be easily identified by the fluctuating curves in Figure 3k (−35–−10 µA) compared to those in Figure 3l (−100–−20 µA). This result may be explained by two points: 1) More photogenerated carriers are excited in the MXene/MoS_2_ structure with increased light intensity. 2) The heterojunction can contribute to separating photoexcited electron–hole pairs and enhancing I_S, which can be verified by the change in I_S accompanying V_PE_ sweeping between zero and −2 V, as shown in Figure 3e. In addition, the device without a heterojunction also demonstrates a V_PE_‐controlled I_P signal, especially under a low light intensity (2 mW cm^−2^) as shown in Figure 3k. We deduce that the reason is that there are photogenerated carriers in the pure MXene layer, and some of them are separated by the external electric field force (under different V_PE_s), thus forming the measured current flow (I_P), which increases from orange to green (in Figure 3k). However, this V_PE_ separation process is limited, since the resolution among these curves is lowered as the light intensity increases from 2 to 10 mW cm^−2^. This means that for a larger number of photoelectron–hole pairs, the external electric field force is not sufficient to separate them, and most of them vanish due to recombination. In contrast, for the device with the MXene/MoS_2_ heterojunction (Figure 3l), there are more clearly discrete and upward‐shifted curves under a higher light intensity (5 mW cm^−2^), making it a good candidate for simulating the circadian neural response.

### 2.3 Serotonin‐Modulated OANS

**Figure 4:**
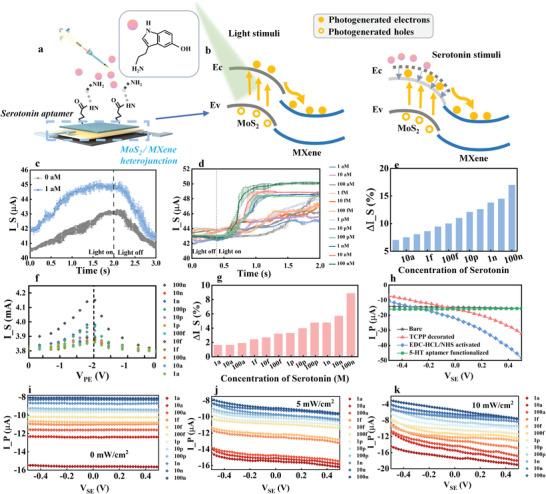
a) The schematic diagram of the OANS device under the dual modulations of light and serotonin. b) The I_S is modulated by increasing serotonin and light excited band bending. c) The on‐off light triggered dynamic I_S curves before and after cross‐linked 1 am serotonin, V_PE_ = −2 V, V_SE_ = 0.1 V and light intensity was 5 mW cm^−2^. d) I_S dynamic curves under on‐off light (5 mW cm^−2^), with the increasing serotonin concentration (1 am–100 nm), V_PE_ = −2 V, V_SE_ = 0.1 V. The error bars represent the standard deviation of randomly selected five individual OANS devices. e) ∆I_S triggered by on‐off light, with increasing serotonin concentration (1 am–100 nm). f) The dynamic SC scanning results with different serotonin concentrations. ∆I_S at the peak points with increased serotonin concentrations are summarized in (g). h) Transfer characteristic curves in the process of surface modification, when V_PE_ = −2.72 V. i–k) Transfer characteristic curves with increased concentrations of serotonin when V_PE_ = −2.72 V, under different light intensities of 0, 5, and 10 mW cm^−2^.

Following the experiments on light modulation of the synaptic plasticity, further studies were conducted on neurotransmitter‐modulated OANS devices. The experimental scheme is illustrated in **Figure** **4**a. The proposed device can convert an optical signal to I_S, and this ability can be modulated by V_PE_ (demonstrated in Figure 3l and diagrammed by the band bending in Figure 4b). Accordingly, if a biochemically modified interface is embedded on the surface of MoS_2_, as in our previous work about the MXene synapse,^[^ ^22^ ^]^ biochemical adjustments of the effective bias voltage on the heterojunction may be realized, resulting in the changed band bending degree (Figure 4b). Then, photochemistry modulation of the synaptic plasticity may be produced. To test this design, the specific aptamer to serotonin was crosslinked on the TCPP decorated MoS_2_, and the molecular formulas of the crosslinking materials are shown in Figures S20 and S21 (Supporting Information). I_S was examined under controlled V_PE_, V_SE,_ and light intensities when different concentrations of neurotransmitters were pipetted. First, the chemical modulation is found by dynamic I_S curves in Figure 4c. The light‐on‐off triggered I_S curves shift upward when the serotonin concentration increases from 0 to 1 am, which means that more carriers are transferred to the conductive filament region even when the same bias voltage and light intensity are applied. Since the only changed condition is the concentration of serotonin, it is deduced that the shifted I_S curves may be attributed to the captured serotonin‐molecule‐induced alteration on the band bending of the heterojunction. Then, the energy consumption during a 10 ms duration time of vision was calculated, and the energy consumption between each neurotransmitter concentration level is only 500 fJ, which is close to the energy consumption (100 fJ) in the signal transmission process of biological synapses. The calculation method is provided in Figure S22 (Supporting Information). Therefore, the proposed OANS device demonstrates the potential to construct low‐power‐consumption circadian neuromorphic devices. The more upshifted I_S curves for the refined concentration intervals in Figure 4d reveal that as the concentrations of serotonin are increased from 1 am to 100 nm, more abrupt I_S changes can be observed, and higher current jumps are triggered, as evaluated by ∆I_S in Figure 4e. This result demonstrates that the resistance states of OANS devices can be successfully adjusted by neurotransmitter concentrations. In addition, the repeated serotonin stimulus testing is shown in Figure S23 (Supporting Information), it can be observed that the synaptic currents exhibit a stepwise ascending when higher and higher serotonin solutions are repetitively exerted. To explore and demonstrate our surmise about the chemical modulation on heterojunction band bending, dynamic tests with V_PE_ sweeping between 0 and −2 V and the serotonin concentrations gradually increasing from 1 am to 100 nm, were conducted under V_SE_ = 0.1 V and on‐off light (5 mW cm^−2^) conditions, as presented in Figure 4f. The elevated scanning I_S curve and the summarized ∆I_S at the peak points in Figure 4g indicates that more carriers are transferred into the conductive filament region. Since the serotonin concentration is the only changed experimental element, we believe that the extra carriers may come from the neurotransmitter‐adjusted heterojunction. It is deduced that the specifically captured serotonin molecules can adjust the bias of the heterojunction and exacerbate its band bending, which consequently allows more electrons to pass through the heterojunction and participate in the formation of I_S. Further examinations of the interfacial influences of the proposed OANS device were conducted by comparing transfer characteristic curves in the process of surface modification, which are given in Figure 4h. The carriers collected by PE (i.e., the I_P curves) are changed in accompanies with TCPP modification, aptamer linking and target neurotransmitter capture. Similar measurements were executed under the same experimental conditions and different serotonin concentrations. The upshifted curves with increasing concentrations in Figure 4i–k demonstrate that more electron–hole pairs are separated by the strengthened band bending, and then, the separated holes are collected by the PE. Correspondingly, the separated electrons are transported to the conductive filament region with the aid of tunneling effect, as measured by the increased I_S curves in Figure 4c,d. Meanwhile, the increased photogenerated carriers can also be demonstrated by comparing the enlarged I_P change ranges with increasing light intensity from 5 (Figure 4j) to 10 mW cm^−2^ (Figure 4k). To verify the potential of the proposed OANS device in artificial neural network applications, the resistance state under 16 different serotonin concentrations (0–100 µm) after light activation is counted in Figure S24 (Supporting Information), which exhibits a stable 4‐bit resistance retention characteristic.

### 2.4 Imitation of Circadian Learnability

**Figure 5:**
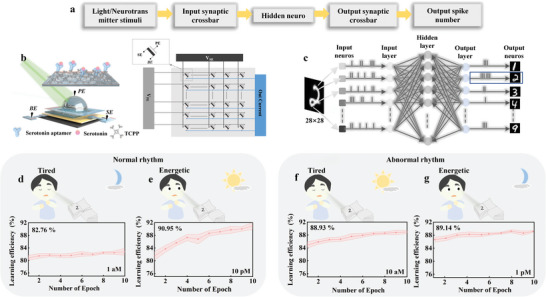
The scenario diagram of the proposed biomimetic OANS to simulate circadian learnability. a) The flow chart of the simulation process. b) Schematic of the hardware implementation of the light and neurotransmitter dual stimulated neural network by using the proposed optic nerve synaptic device at each intersection. c) The schematic of the spiking neural network (SNN) for MNIST classification. It contains 784 input neurons (OANS device), 100 hidden neurons, and ten output neurons. The two learning efficiency results related to the normal circadian rhythm behaviors, which are d) Tired at night, and e) Energetic during the day. The two learning efficiency results are related to the abnormal circadian rhythm behaviors, which are (f) Tired during the day and (g) Energetic at night. The error bars represent the standard deviation of randomly selected five individual OANS devices.

Neurological studies have revealed that human learning and memory have the characteristics of circadian rhythm, and OANS plays a key role in maintaining circadian rhythm.^[^ ^43^, ^44^ ^]^ In this section, the experiments simulating circadian learnability were conducted using the developed biomimetic OANS device, as shown in the flow chart of **Figure** **5**a and described below. The external stimuli applied to the biomimetic OANS are perceived by the input synaptic crossbar. The biomimetic OANS is positioned at each cross point (as shown in Figure 5b) to produce neural responses to learning objects (images of handwritten digits, as a proof‐of‐concept) under different light intensities and serotonin concentrations. These stimuli are analogous to the environmental day–night optical conditions and the biological endocrine system. The conductivity variations of the device under different concentrations are measured multiple times and used to simulate the weight matrices. The average Set and Reset voltages are used to simulate the threshold value of the leaky integrate and fire (LIF) neuron. The MNIST database is used as simulation input for SNN training, which consists of a large number of handwritten digital pictures with 28 × 28 binary pixels. And they are split into two sets, one is for network training another is for testing. The training set contains 60 000 images and the testing set has 10 000 images. Then, the collected I_S data are input to a three‐layer SNN and filtered through a brain‐like analysis to output a “recognized” digit (Figure 5c). The learning efficiency of the proposed OANS is assessed by using MNIST handwritten digit classification results; that is, the learning efficiency is assessed by the mean value and standard deviation of the recognition accuracy of 5 randomly selected individual OANS devices. Figure 5d–g and Figure S25 (Supporting Information) are the scenario diagrams of the proposed biomimetic OANS to simulate circadian learnability. The “Boy” with a normal rhythm in Figure 5d,e is tired at night and has relatively lower learning efficiency (82.76%). This phenomenon is simulated without light and with a small amount of serotonin (Figure 5d). During the daytime (Figure 5e), he has higher learning efficiency (90.95%). In contrast, the “Boy” with an abnormal rhythm is tired during the daytime (88.93% in Figure 5f) and has a relatively high learning efficiency at night (89.14% in Figure 5g). This abnormal rhythm is stimulated by modulating the light and serotonin content. The simulations also indicate that the learning efficiency in the energetic state of abnormal rhythm (89.14% in Figure 5g) is lower than that of normal rhythm (90.95% in Figure 5e); that is, the energetic state, as a key factor in determining learning efficiency, is affected by circadian rhythm, which is consistent with neurobiology research.^[^ ^45^ ^]^ Therefore, it is believed that with the use of the proposed biomimetic OANS devices and SNN, the simulation of day–night rhythm learning behavior is accomplished.

## 3 Conclusion

In summary, a novel biomimetic OANS device is proposed and demonstrated to mimic circadian learnability. First, fundamental experiments on the MXene/MoS_2_ heterojunction‐based biomimetic OANS device confirm that synaptic plasticity can be modulated by controlled illumination and serotonin stimuli, which are analogous to environmental light and internal secretion. The interpretations of these interesting double modulations observed in the proposed OANS device are provided according to the classical theories of conductive filament and tunneling effects. Second, the simulation of two kinds of circadian rhythmic behaviors (normal and abnormal rhythms) is implemented by integrating the proposed OANS devices with a three‐layer SNN to produce light and neurotransmitter dual‐modulated synaptic responses, similar to the visual sensation and learning processes in the nerve center. Circadian learnability can be successfully imitated by the proposed OANS, which indicates that this work may provide new possibilities for developing biomimetic OANS. In the future, such a complete nervous system may be expected to be included in neuromorphic intelligent robots.

## 4 Experimental Section

### Biomaterials

BSA was purchased from Genview, USA. TCPP, 1‐ethyl‐3‐(3‐dimethylaminopropyl) carbodiimide hydrochloride (EDC·HCl), N‐hydroxysuccinimide (NHS), cortisone, and corticosterone were procured from Shanghai Yuanye Biological Technology Co., Ltd., China. The serotonin aptamer is a 5′‐NH_2_‐modified oligonucleotide with a sequence of 5′‐NH_2_‐(CH_2_)_6_‐CGA CTG GTA GGC AGA TAG GGG AAG CTG ATT CGA TGC GTG GGT CG‐3′ was purchased from Shanghai Sangon Biotech, China.

### Synthesis of the MXene Ti_3_C_2_T_x_ Nanosheets

First, 0.333 g of HF, 2.5 mL of 12 m hydrochloric acid, and 0.5 g of Ti_3_AlC_2_ powder were mixed; then 2.5 mL of deionized water was added to the mixture and purged with N_2_ for 20 min to replace oxygen. After stirring for 24 h in a silicon oil bath (40 °C), the resulting solution was centrifuged (4000 rpm) in 40 mL of deionized water for 5 min. After five times centrifugation, the obtained material was dried in a constant temperature drying oven at 80 °C for 24 h. The multilayer Ti_3_C_2_T_x_ nanosheets were obtained. Ultrasonic dissociation for 10 h after adding 100 mg multilayer Ti_3_C_2_T_x_ nanosheets to 80 mL deionized water. Subsequently, the mixture was centrifuged at 4000 rpm for 10 min. After the centrifugation, the supernatant was collected to obtain a uniform few‐layer Ti_3_C_2_T_x_ nanosheet solution.

### Synthesis of the MoS_2_ Nanosheets

The bulk MoS_2_ (0.05 g) was put into the prepared DES ionic solution (a solution of acetylcholine and propanediol with a molar mass ratio of 1:2). Then, the sample was heated and stirred for 6 h in a water bath (60 °C). The expanded MoS_2_ was then centrifuged in deionized water at 4500 rpm two times, and pure expanded MoS_2_ was obtained as a solid precipitate. The expanded MoS_2_ powder was then placed into a 10 mL BSA solution (1 mg mL^−1^). BSA was not only an effective exfoliating material but also a strong stabilizer preventing the reaggregation of nanosheets, which greatly improves their biocompatibility. This solution was sonicated for 20 h. Then, the MoS_2_ and BSA solutions were completely separated by centrifuging at 15 000 rpm three times. The collected precipitate was dispersed in deionized water by sonication for 10 min. The resulting solution was then centrifuged at 1500 rpm three times. The obtained supernatant contained the MoS_2_ nanosheets.

### Fabrication of OANS Device

1) The indium tin oxide (ITO) glass was used as the BE. The MXene Ti_3_C_2_T_x_ and MoS_2_ heterojunction film were prepared by regular spin‐coating method layer by layer. 2) The patterned Ag electrodes were constructed by the microelectronic printer (Shanghai Mifang Electronic Technology Co., LTD, China.). 3) The insulating ink was used to insulate and encapsulate the prepared OANS. (4) The neurotransmitter sensing area was constructed on the MXene/MoS_2_ heterojunction film by using the PBS buffer solution.

### Biofunctionalization of Serotonin Aptamer

The fabricated devices were biofunctionalized by the conventional method. 1) 20 µL of TCPP was dropped and incubated for 2 h at room temperature and then rinsed off with deionized water, the central macrocycle of TCPP can be attached to the MoS_2_ layer through the π─π* stacking, and the carboxyl groups around the macrocycle can be used to covalently anchor the aptamer probe.^[^ ^46^, ^47^ ^]^ 2) TCPP‐modified devices were treated with EDC·HCl (200 mm) and NHS (50 mm) to activate the carboxyl groups at room temperature for 30 min.^[^ ^48^ ^]^ 3) 50 µL of the serotonin aptamer solution was incubated overnight at 4 °C to make the probes covalently linked onto the TCPP‐modified OANS device because the four carboxyl groups of TCPP are beneficial to increasing the density of the aptamer.^[^ ^47^ ^]^ 4) 50 µL of 0.5% BSA was used to block the residual carboxylate groups. 5) 20 µL of the serotonin sample was dropped for the test.

### Apparatus

Transmission electron microscopy (TEM) and high‐resolution TEM (HRTEM) examinations were carried out by using a Tecnai G2 F20 (FEI, USA). Scanning electron microscopy (SEM) images were obtained by using a Quanta 200 (FEI, USA). All electrical measurements were performed by using an Agilent semiconductor parameter analyzer.

### Code Availability

The code used for the simulations is the open‐source code of Duan et al. (DOI: https://doi.org/10.1038/s41467‐020‐17215‐3).^[^ ^49^ ^]^

### Statistical Analyses

All error bar results were performed in five independent experiments and described in the Figure legends. Statistical analyses were performed using Origin 2021 software.

## Conflict of Interest

The authors declare no conflict of interest.

## Supporting information

Supporting Information

## Data Availability

The data that support the findings of this study are available from the corresponding author upon reasonable request.
