# Supplementary material for: An Ultrasensitive Biomimetic Optic Afferent Nervous System with Circadian Learnability
Source: Adv Sci (Weinh). 2024 Mar 11;11(21):2309489. doi: 10.1002/advs.202309489 (PMC11151074; doi:10.1002/advs.202309489)
Supplement: Supplementary file 1 — Supporting Information [file ADVS-11-2309489-s001.pdf]

ADVANCED SCIENCE
Open Access

Supporting Information

for *Adv. Sci.*, DOI 10.1002/advs.202309489

An Ultrasensitive Biomimetic Optic Afferent Nervous System with Circadian Learnability

Kaiyang Wang, Shuhui Ren, Yunfang Jia and Xiaobing Yan**

Supporting Information

An ultrasensitive biomimetic optic afferent nervous system with circadian learnability

Kaiyang Wang,[1‡] Shuhui Ren,[1‡] Yunfang Jia,[1*] Xiaobing Yan.[2*]

[1]*College of Electronic Information and Optical Engineering, Nankai University, Tianjin, 300071, P. R. China*

[2]*Key Laboratory of Brain-Like Neuromorphic Devices and Systems of Hebei Province, College of Electron and Information Engineering, Hebei University, Baoding 071002, P. R. China*

**Correspondence should be addressed to Y. Jia (email: jiayf@nankai.edu.cn), X. Yan (email: yanxiaobing@ime.ac.cn)*

[‡]*K.W. and S.R. contributed equally to this work.*

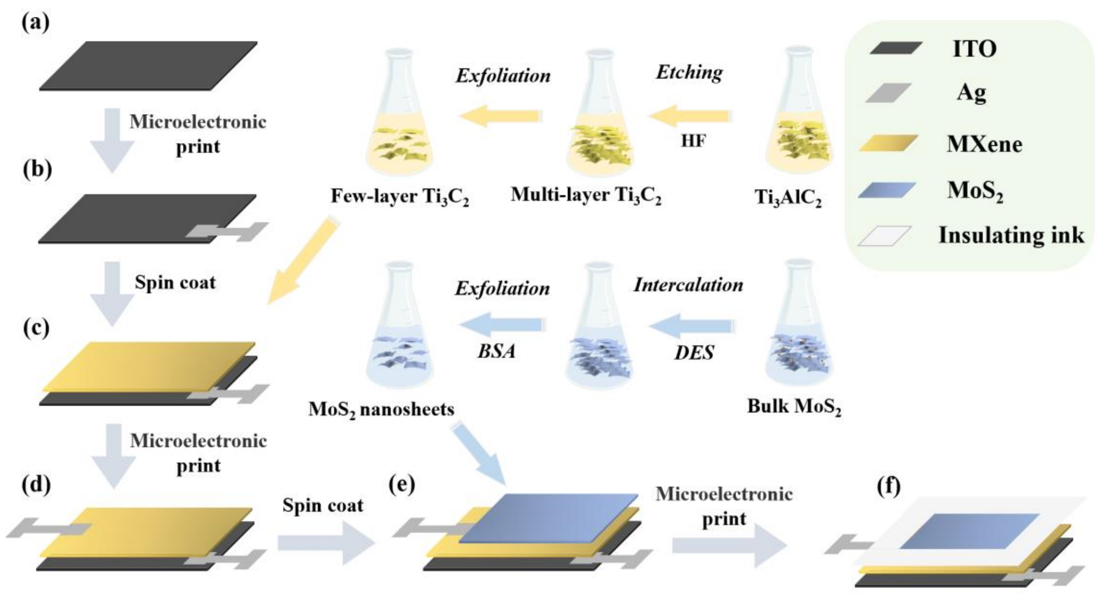

Fig. S1 Diagrams for preparing the proposed optic afferent nervous system (OANS) device. (a) The indium tin oxide (ITO) glass is used as the bottom electrode. (b) After microelectronic printing, the bottom electrode is constructed. (c) The MXene film is spin coated on ITO. (d) After the second microelectronic printing, the Ag synapse electrode (SE) is constructed on the one side of MXene film. (e) Then the MoS_2 film is spin coated on the MXene film to form the heterojunction. (f) The insulating ink is used to insulate and encapsulate the prepared OANS.

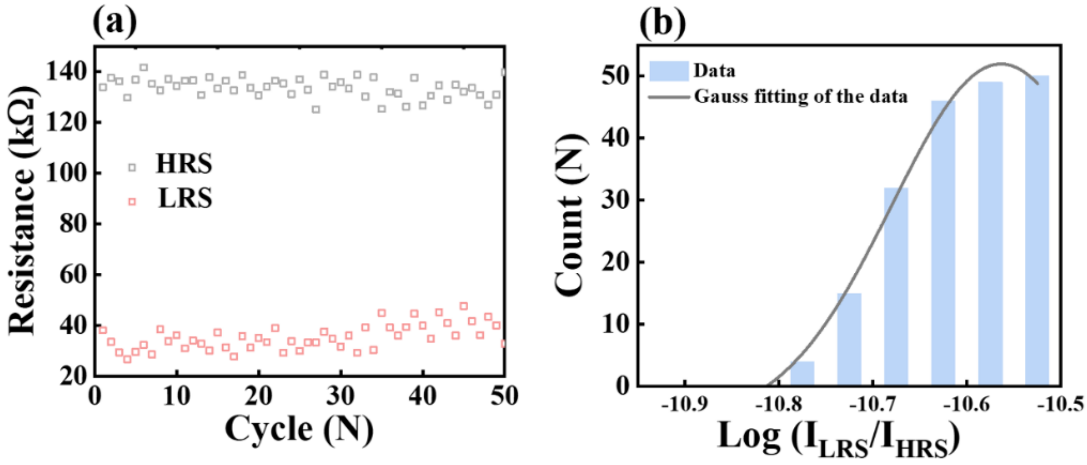

Fig. S2 The basic electronic performance of MXene $Ti_3C_2T_x$ artificial synapse. (a) The statistics of resistance in high resistance state (HRS) and low resistance state (LRS). (b) Gauss fitting result of the current ratio in LRS and HRS.

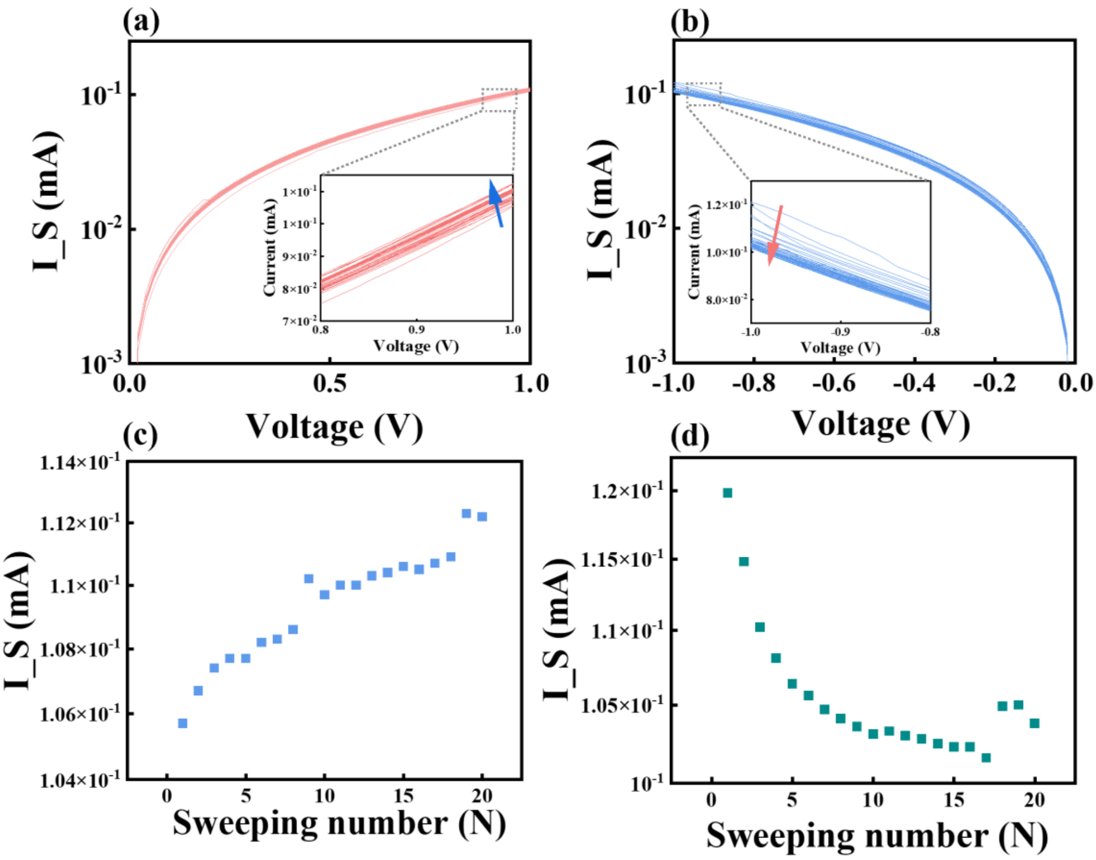

Fig. S3 Asymptotic nonlinear I-V curves under the positive (a) and negative (b) voltages applied on the synapse electrode (SE), the blue (a) and red (b) arrows designate the moving trends of synaptic currents (I_S) with the increased sweeping times. The stabilized I_S after each of the sweeping cycles under positive (c) and negative (d) voltages.

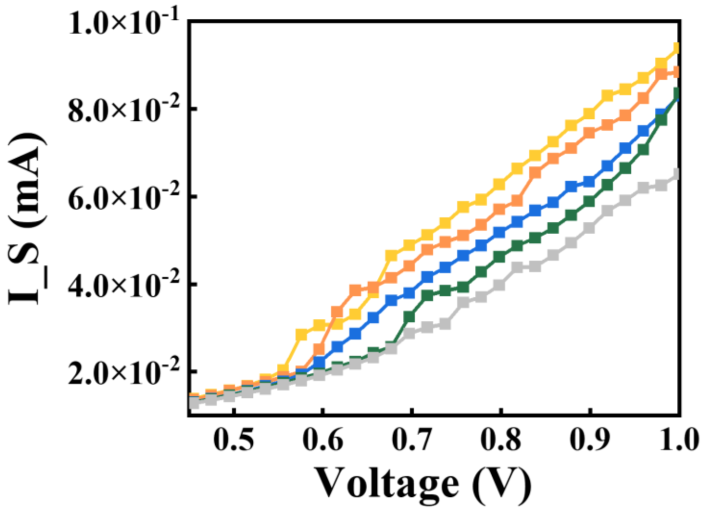

Fig. S4 The enlarged CV curves of the proposed device, when V_{PE} is 0 V (gray). -0.5 V (green), -1.0 V (blue), -1.5 V (orange), and -2.0 V (yellow), respectively.

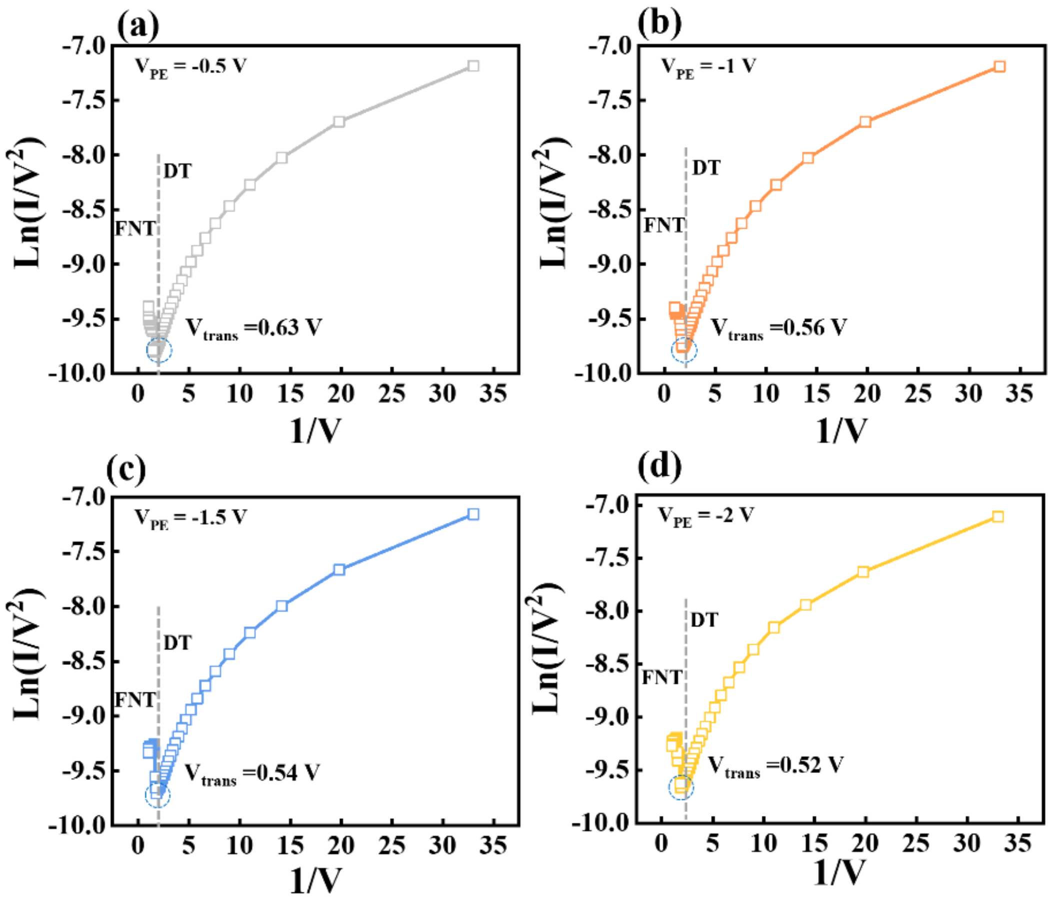

Fig. S5 The I-V fittings with $\ln(I/V^2) \propto 1/V$ for the artificial synapse in the difference V_{PE} indicate the FNT and DT mechanism.

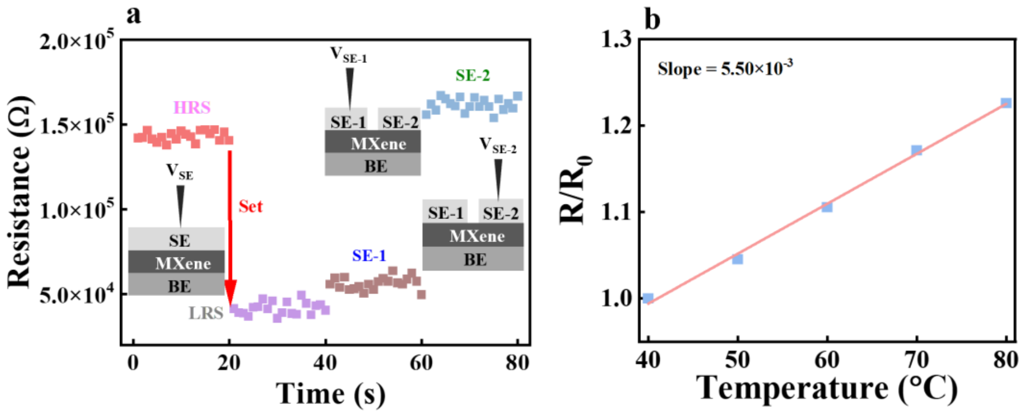

Fig. S6 a. Electrode cutting test. b. Temperature dependence test.

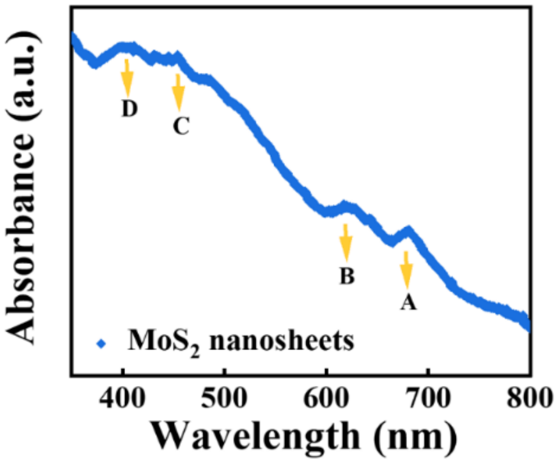

Fig. S7 The UV absorption spectrum of the MoS_2 nanosheets. It is indicated that the MoS_2 nanosheets partially absorb light in the visible range. There are four typical absorption peaks (A, B, C and D) which reflect the semiconductor behavior of the MoS_2 nanosheets.

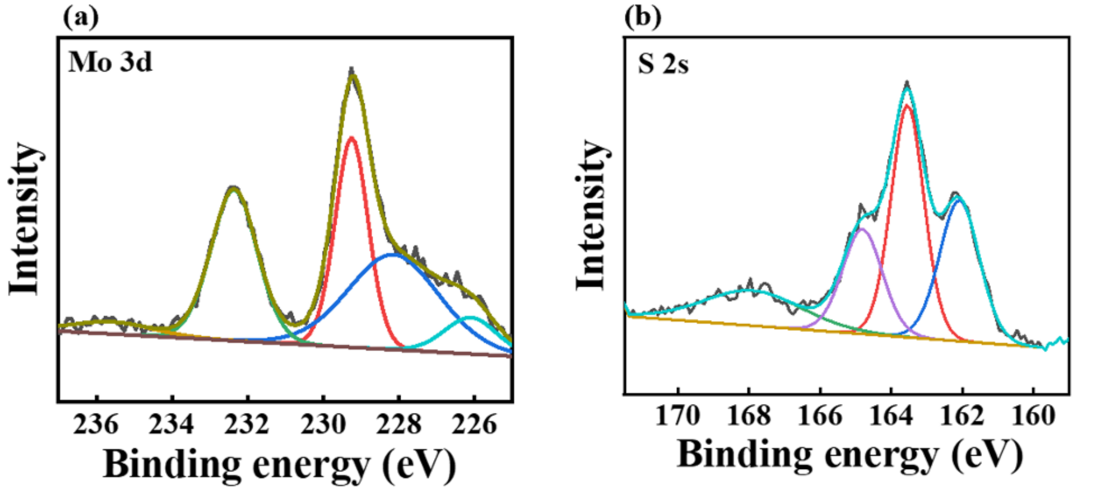

Fig S8 The XPS spectra of exfoliated MoS_2 nanosheets. (a) Mo 3d. (b) S 2s. (C) XPS full spectrum. The two strong peaks in **Fig. S8(a)** at 229.2 and 232.3 eV, respectively, demonstrated the 2H phase absorption peaks of the MoS_2 nanosheets. Then the two weak absorption peaks at 226.2 and 228.4 demonstrated the 1T phase in the MoS_2 nanosheets. In the S 2p region of the spectra (**Fig. S8(b)**), additional peaks are found beside the known doublet peaks of 2H phase MoS_2, S $2p_{1/2}$ (166.3), and S $2p_{3/2}$ (164.6), which appear at 163.0 eV and 162.1 eV, respectively. The change of these peaks can be ascribed to the formation of the 1T and 2H phase. The deconvolution analysis of Mo and S XPS peaks reveal the used MoS_2 nanosheet is 1T/2H mixed phase.

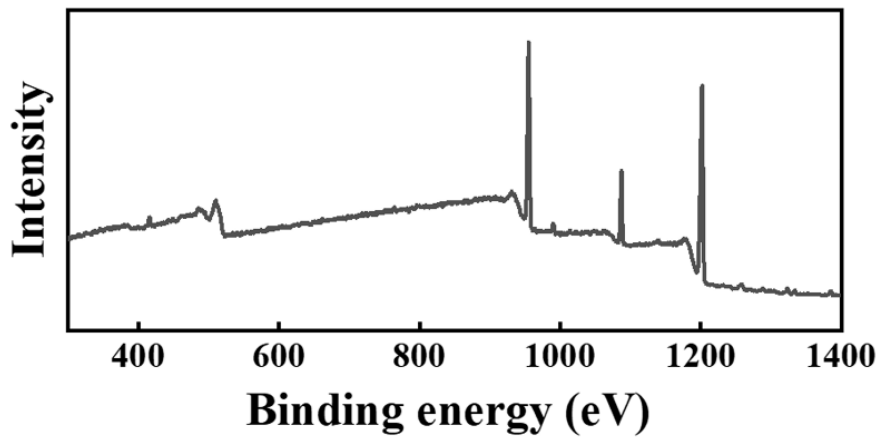

Fig. S9 The XPS full spectrum of exfoliated MoS_2 nanosheets.

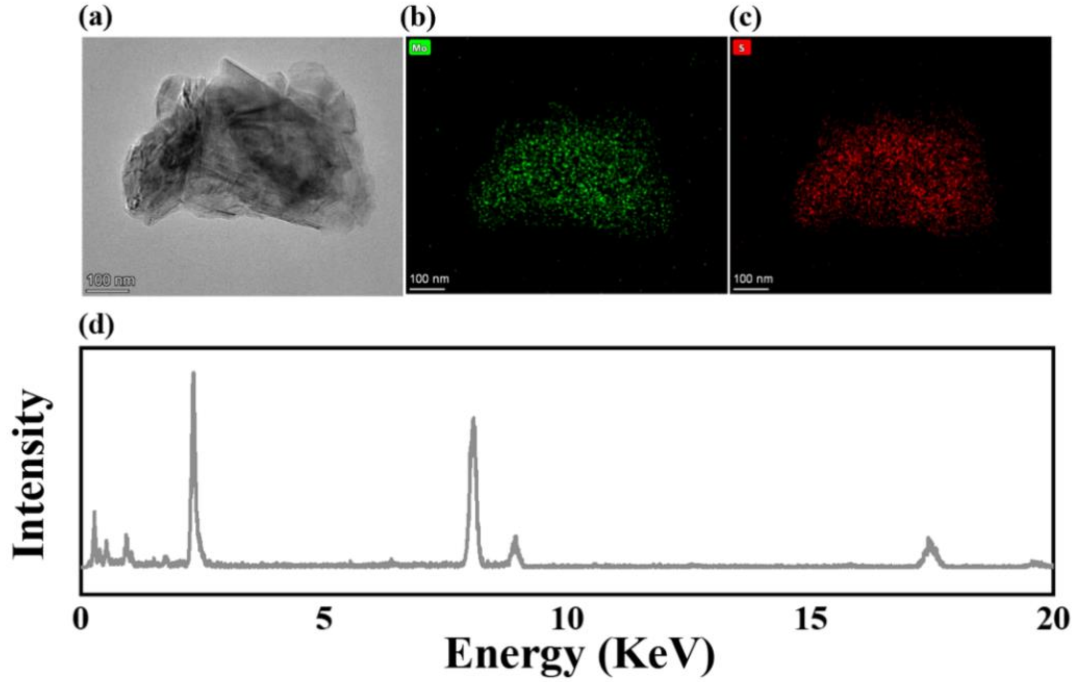

Fig. S10 The high-resolution transmission electron microscope (HRTEM) image of the MoS_2 nanosheets. (a) HRTEM image of an individual MoS_2 nanosheet, its size is about 800 × 500 nm, which is helpfully used for the spin-coating method to form the MoS_2 film. (b) - (d) The element mapping information of the Mo and S.

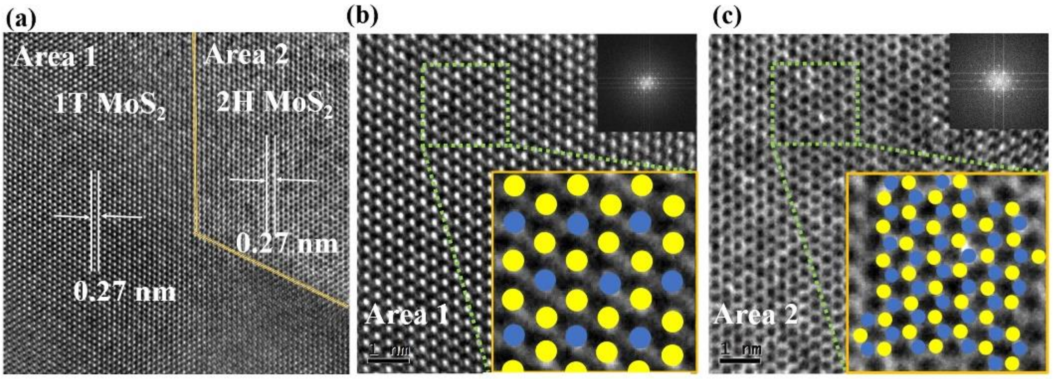

Fig. S11 The HRTEM crystal phase analysis of the MoS_2 nanosheets. (a) 1T and 2H phase of the prepared MoS_2 nanosheets. (b) and (c) 1T and 2H phase structure diagram. Remarkably, there are two distinct lattice regions in a plane, the hexagonal lattice region with crystalline space of 0.27 nm is indexed to 2H MoS_2, and the lattice fringe spacing of trigonal lattices region with 0.27 nm crystalline space ascribed to 1T MoS_2.

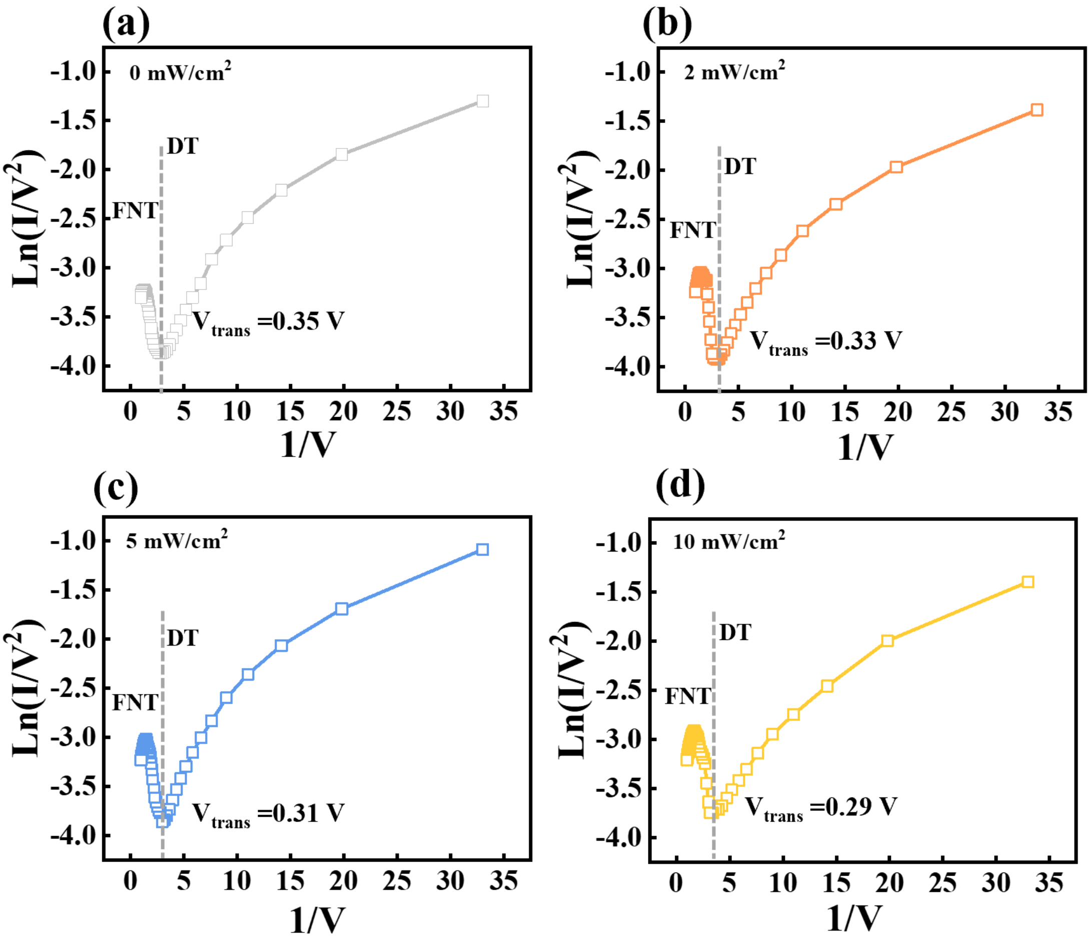

Fig. S12 The I-V fittings with $\ln(I/V^2) \propto 1/V$ for the artificial synapse under the different light intensity indicate the FNT and DT mechanism.

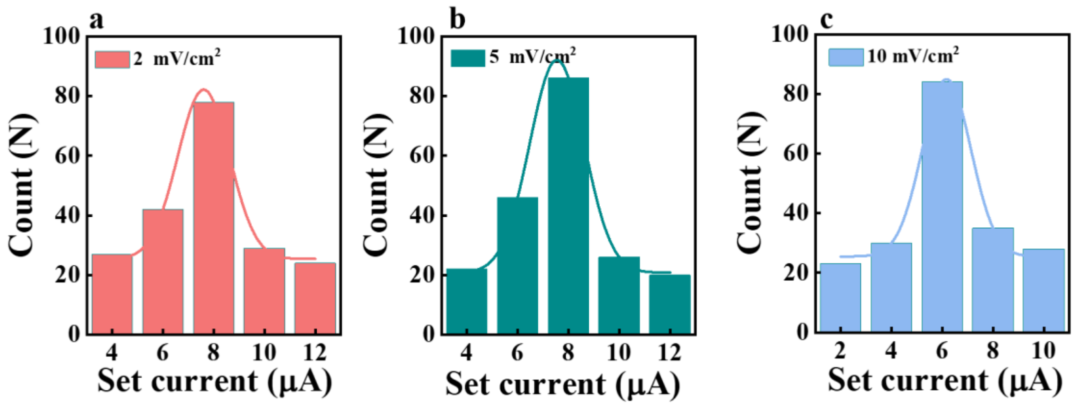

Fig. S13 The Gauss statistical analyses of Set current under different light intensities.

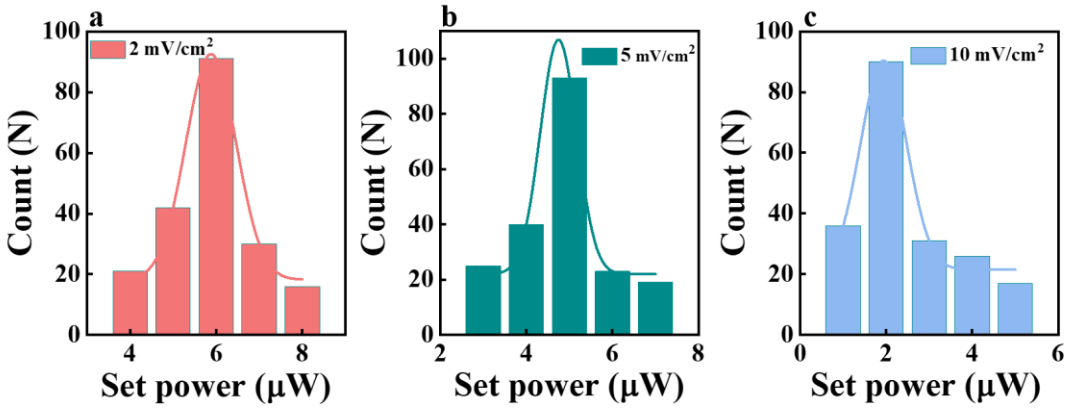

Fig. S14 The Gauss statistical analyses of Set power under different light intensities.

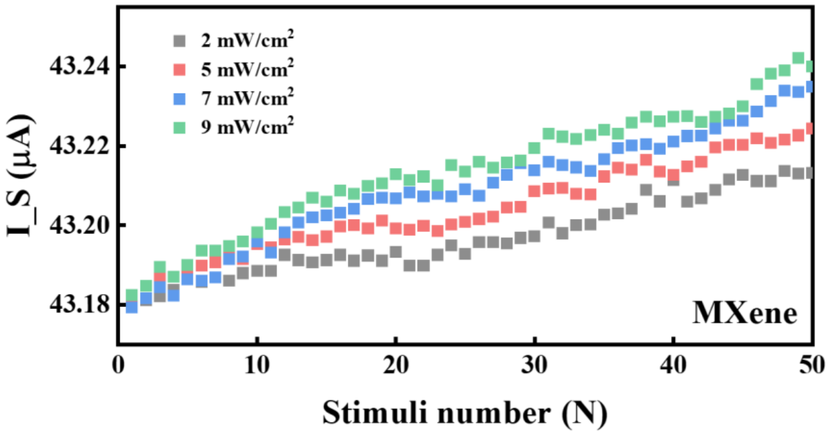

Fig. S15 The dynamic I_S transient curve of the device without MoS_2/MXene heterojunction in response to 2 - 9 mW/cm^2 light, V_{SE}= 0.1 V, V_{PE}= -2 V.

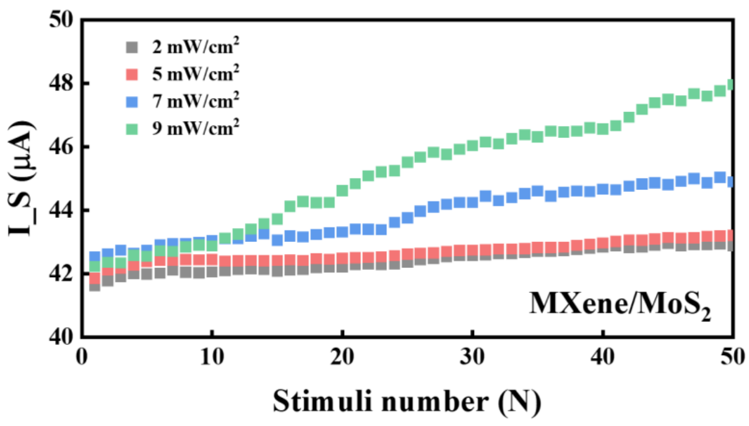

Fig. S16 The dynamic I_S transient curve of the device with MoS_2/MXene heterojunction in response to 2 - 9 mW/cm^2 light, V_{SE}= 0.1 V, V_{PE}= -2 V.

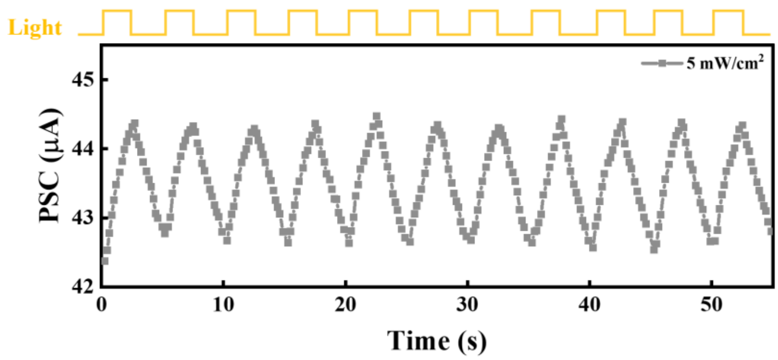

Fig. S17. The cyclic responses to repeated light stimuli.

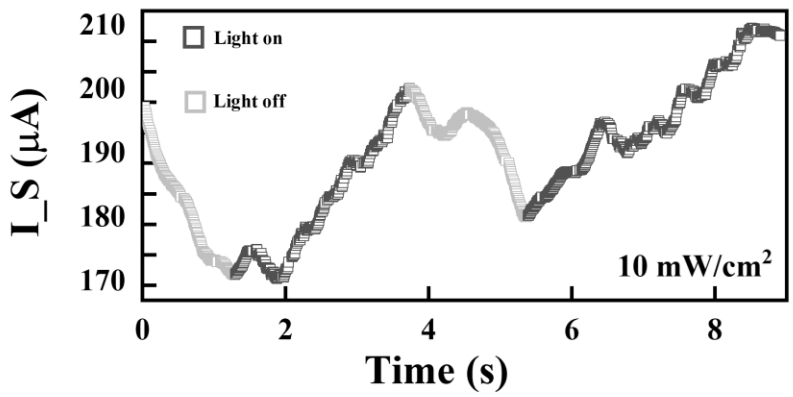

Fig. S18 The dynamic I_S transient curve of the device with MoS_2/MXene heterojunction in response to 10 mW/cm^2 light, V_{SE}= 0.1 V, V_{PE}= -2 V.

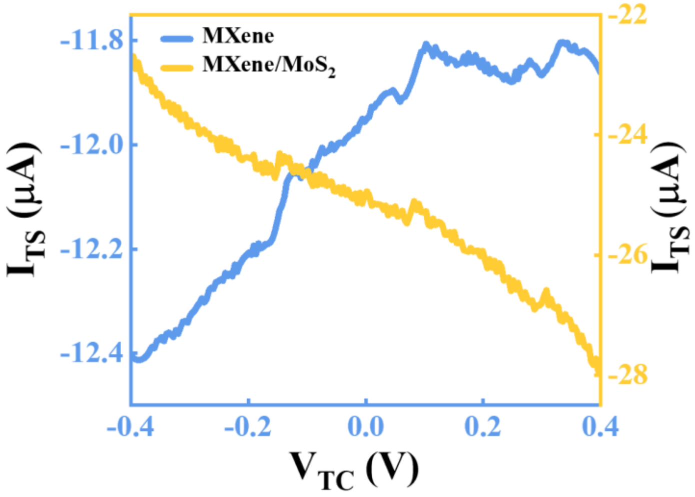

Fig. S19 The transfer characteristic curves of the as-prepared devices with and without heterojunction MXene/MoS_2.

(a)

EDC-HCL

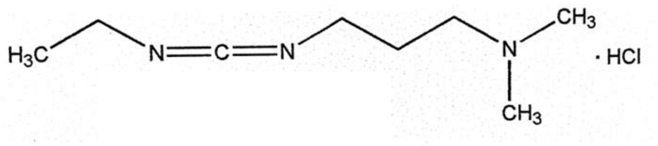

(b)

NHS

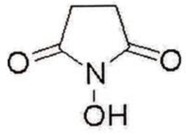

Fig. S20 The molecular formulas of EDC-HCL and NHS.

TCPP

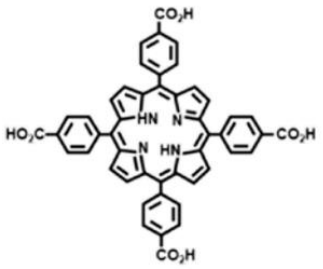

Fig. S21 The molecular formula of TCPP.

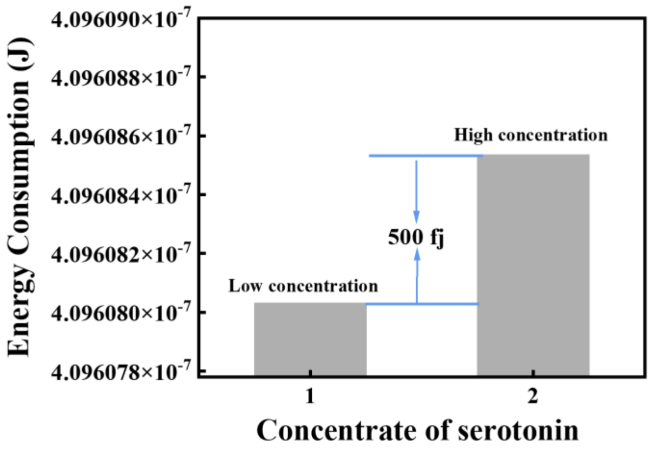

Fig. S22 The energy consumption of the proposed OANS device from low concentration to high concentration, in which the energy consumption is 500fj.

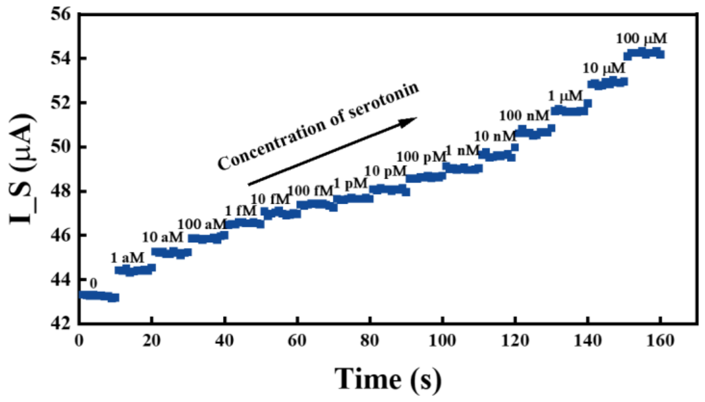

Fig. S23 The I_S of the OANS device with the increased concentrations of serotonin.

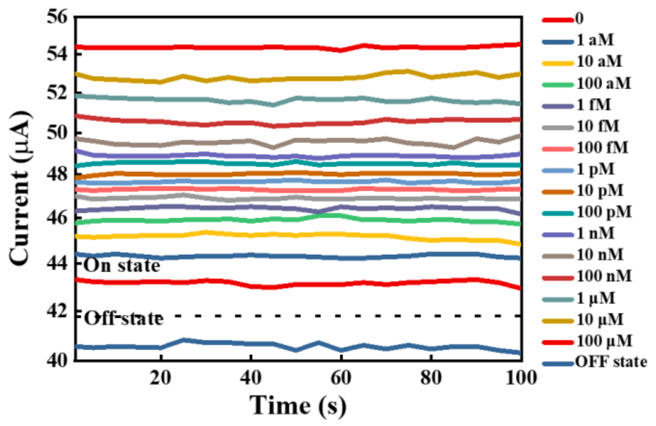

Fig. S24 Multilevel resistance retention characteristic under different serotonin concentration.

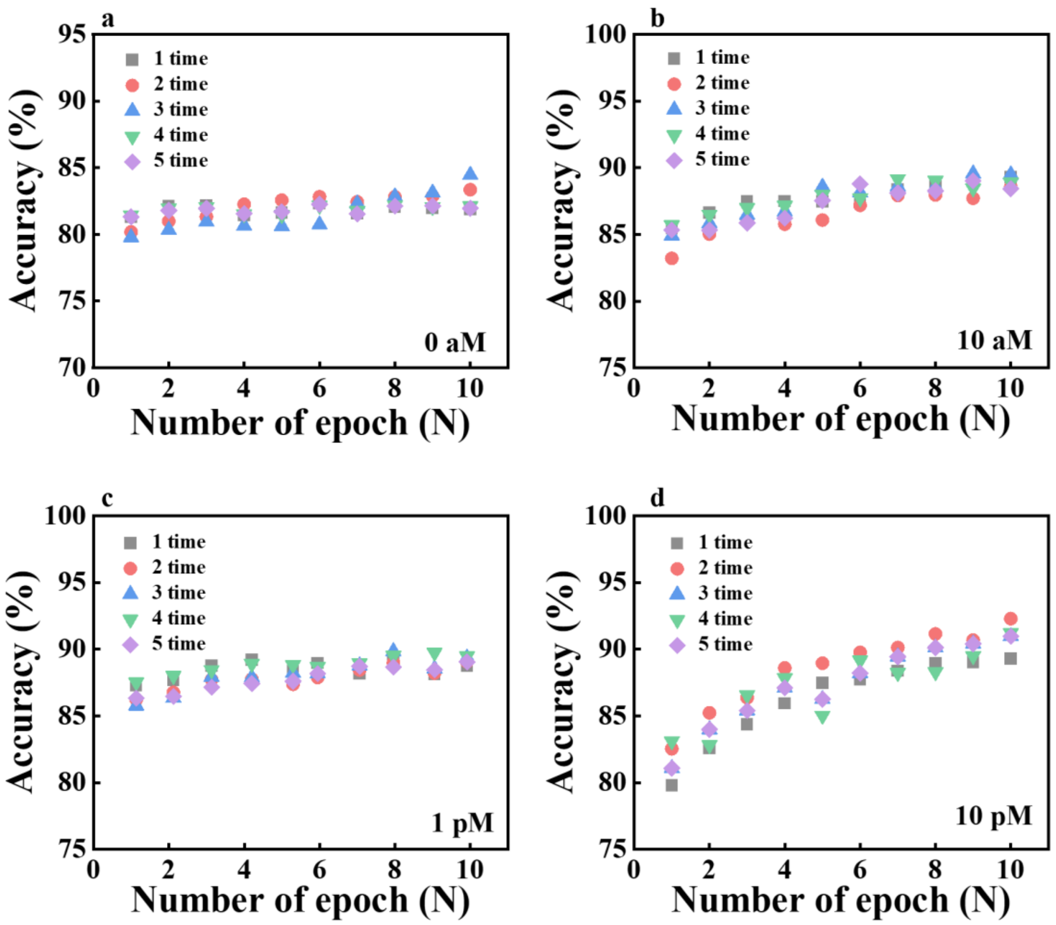

Fig. S25 The 5 times learning efficiency results of the proposed OANS device under different concentrations (0 aM, 10 aM, 1 pM, 10 pM) of serotonin.

Table 1. Compare the electronic performances with 2D material-based devices.

**Device type**	**Device name**	**Set current (A)**	**Set power (W)**	**Set voltage (V)**	**LOD**	**Journal**
	OANS device	~1.5× 10^{-7}	~7 × 10^{-6}	~0.45	**1 aM**	This work
	Chemically neuron	~1×10^{-7}	~0.65 × 10^{-7}	~0.65	1 μM	Nat. Electronics 2022, 5, 586
**Sensing neuromorphic**	Biohybrid synapse	/	/	/	60 mM	Nat. Mater. 2020. 19, 969
	Dual-Gated Organic	/	/	/	10 nM	Adv Mater. 2021, 33,

**devices**	Synapse					2100119
	Organic synapse	/	/	/	1 PM	ACS Sens. 2017, 2, 12, 1756
**2D material-based devices**	$Ag/CdPS_3/ITO$	~10^{-5}	~10^{-5}	~1	/	Adv. Funct. Mater. 2023, 33, 2211269.
	$Cu/HfO_x/BP/Pt$	~5×10^{-5}	~5×10^{-5}	~0.7	/	Adv. Mater. 2023, 2300446.
	Gr/MoS_2–xOx/Gr	~10^{-5}	~10^{-5}	~1.1	/	Nat. Electron. 2018, 1, 130.
	$Ag/MoS_2/Ag$	~10^{-3}	~10^{-4}	~0.66	/	Nano Lett. 2016, 16, 572.
	$Ti/MoTe_2/Au$	~10^{-4}	~2×10^{-4}	~2.3	/	Nat. Mater. 2019, 18, 55.
	$Cu/MoS_2/Au$	10^{-4}	2×10^{-5}	0.25	/	Nano Lett. 2019, 19, 2411.
	Ti/Mo-ReS_2/Ti/Au	~10^{-5}	~10^{-5}	~3	/	Adv. Mater. 2022, 34, 2202722.
	$Cu/MoSe_2$: $CuSe_x/Au$	~10^{-6}	~2×10^{-6}		/	ACS Nano. 2023, 17, 1, 84
